# Effects of HIFU induced cavitation on flooded lung parenchyma

**DOI:** 10.1186/s40349-017-0099-6

**Published:** 2017-08-07

**Authors:** Frank Wolfram, Georg Dietrich, Carsten Boltze, Klaus Vitold Jenderka, Thomas Günther Lesser

**Affiliations:** 1 Department of Thoracic and Vascular Surgery, SRH Wald-Klinikum Gera, Teaching Hospital of Friedrich-Schiller University of Jena, Gera, Germany; 2 Gampt Ultrasonic Solutions mbH, Merseburg, Germany; 3 Institute of Pathology, SRH Wald-Klinikum Gera, Teaching Hospital of Friedrich-Schiller University Jena, Gera, Germany; 4 Institute of Physics and Ultrasound, University of applied science HOME, Merseburg, Germany

**Keywords:** Lung flooding, High intensity focused ultrasound, HIFU, Cavitation

## Abstract

**Background:**

High intensity focused ultrasound (HIFU) has gained clinical interest as a non-invasive local tumour therapy in many organs. In addition, it has been shown that lung cancer can be targeted by HIFU using One-Lung Flooding (OLF). OLF generates a gas free saline-lung compound in one lung wing and therefore acoustic access to central lung tumours. It can be assumed that lung parenchyma is exposed to ultrasound intensities in the pre-focal path and in cases of misguiding. If so, cavitation might be induced in the saline fraction of flooded lung and cause tissue damage. Therefore this study was aimed to determine the thresholds of HIFU induced cavitation and tissue erosion in flooded lung.

**Methods:**

Resected human lung lobes were flooded ex-vivo. HIFU (1,1 MHz) was targeted under sonographic guidance into flooded lung parenchyma. Cavitation events were counted using subharmonic passive cavitation detection (PCD). B-Mode imaging was used to detect cavitation and erosion sonographically. Tissue samples out of the focal zone were analysed histologically.

**Results:**

In flooded lung, a PCD and a sonographic cavitation detection threshold of 625 *Wcm* ^− 2^(*p*_*r*_ = 4, 3 *MPa*) and 3.600 *Wcm* ^− 2^(*p*_*r*_ = 8, 3 *MPa*) was found. Cavitation in flooded lung appears as blurred hyperechoic focal region, which enhances echogenity with insonation time. Lung parenchyma erosion was detected at intensities above 7.200 *Wcm* ^− 2^(*p*_*r*_ = 10, 9 *MPa*).

**Conclusions:**

Cavitation occurs in flooded lung parenchyma, which can be detected passively and by B-Mode imaging. Focal intensities required for lung tumour ablation are below levels where erosive events occur. Therefore focal cavitation events can be monitored and potential risk from tissue erosion in flooded lung avoided.

**Electronic supplementary material:**

The online version of this article (doi:10.1186/s40349-017-0099-6) contains supplementary material, which is available to authorized users.

## Background

Focused Ultrasound Surgery (FUS) using High Intensity Focused Ultrasound (HIFU) represents a complete non-invasive modality for local thermal tumour ablation without damages to the surrounding parenchymal tissue [1]. In clinical and pre-clinical trials, HIFU treatment has shown its therapeutic efficacy in many organs, such as for prostate, uterus, breast, liver, brain and many more [2, 3]. In animal models we could demonstrate that FUS treatment of lung tumours is feasible using One Lung Flooding (OLF) [4]. During OLF, the intrapulmonary gas content of one lung wing section is replaced with saline, while the contralateral lung maintains its ventilation. In the flooded condition, unhindered acoustic access to lung tumours for diagnostic [5] and therapeutic ultrasound [6] is given, thus, enabling USgFUS of central lung tumours or targets in adjacent organs by using trans pulmonary HIFU [7]. OLF sounds invasive, but its safety has been shown on large animal models, causing neither disturbance of oxygenation and hemodynamic or surfactant outwash [8, 9].

Cavitation is an acoustic effect in therapeutic ultrasound fields. It is characterized by the appearance of vapour and gas bubbles that oscillate or collapse under the influence of the acoustic pressure field. For monitoring cavitation events, several techniques can be used. Cavitation clouds appear sonographically as hyper-echoic regions [10]. Additionally, the oscillation behaviour of bubbles is associated with hyper-and subharmonic emissions, which can be monitored passively (PCD) [11]. It has been found that subharmonics correlate well with tissue erosion [12]. In general, cavitation bubbles have a high thermal and mechanical activity, and are exposed to the radiation force of the insonating HIFU field, therefore inducing a high stress that can damage tissue mechanically. It has been shown that cavitation can accelerate the thermal energy deposition in tissue [13], which is used for histotripsy [14]. The threshold of cavitation induction and the stress to tissue is dependent upon many factors, such as atmospheric pressure, temperature, gas content and physical properties including surface tension and viscosity [15]. Because of the nonlinear influence of many parameters including HIFU pulse duration and duty cycle, it is difficult to estimate cavitation thresholds numerically.

It has been noticed that cavitation cause unwanted damage of healthy parenchyma and haemorrhage. The effect of vascular tissue erosion caused by cavitation was addressed early as a possible adverse event during HIFU [16]. However, the focus should be targeted during a clinical FUS treatment into tumour tissue where cavitation is not undesired, but in cases of misalignment and in the pre-focal zone, parenchyma is exposed to acoustic pressure and can cause unwanted damage to overlying or adjacent parenchymal tissue.

It is likely that in flooded lung HIFU, cavitation will be induced easily due to its high saline content. Because of the untypical nature of flooded lung as a water-tissue compound, the thresholds of cavitation detection and erosive effects might be different than in known parenchymal tissue, such as liver tissue. Therefore the aim of this study was to determine detection thresholds for HIFU induced cavitation and erosion in flooded lung parenchyma.

## Methods

### Sample selection and preparation

**Fig. 1:**
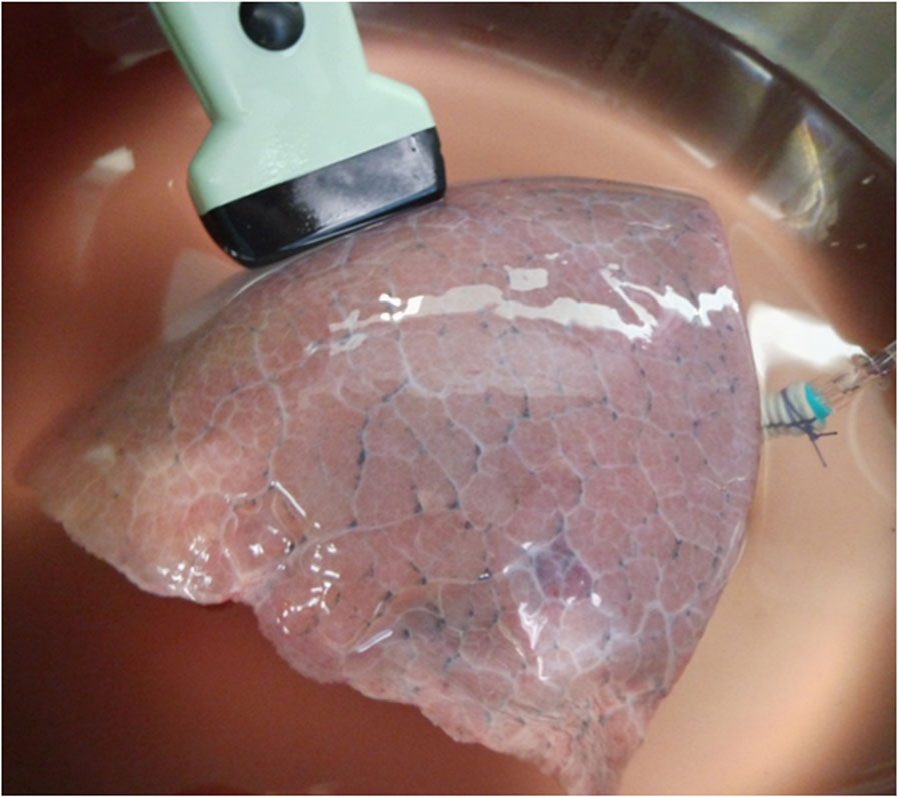
Model of the *ex-vivo *human lung lobe after flooding with saline

Human lung lobes from surgery were used for this study. The *ex-vivo* lung model generates the same quality of gas free lung parenchyma as in-vivo models and is described detailed elsewhere [6]. The samples were resected under curative intent treatment from lung cancer patients. Immediately after resection, flooding was performed with tempered (35 °C), degassed saline (0,9%) until a static pressure of 20 cm water column was achieved. The flooding was qualified by sonography using a portable imaging system (Sonosite Inc., Bothell, WA, USA) with high frequency linear probes (Fig. 1). In total, seven flooded human lung lobes (47–73y, mean 61y) were used (3 upper, 1 middle, 3 lower) in this study.

### Experimental setup

**Fig. 2:**
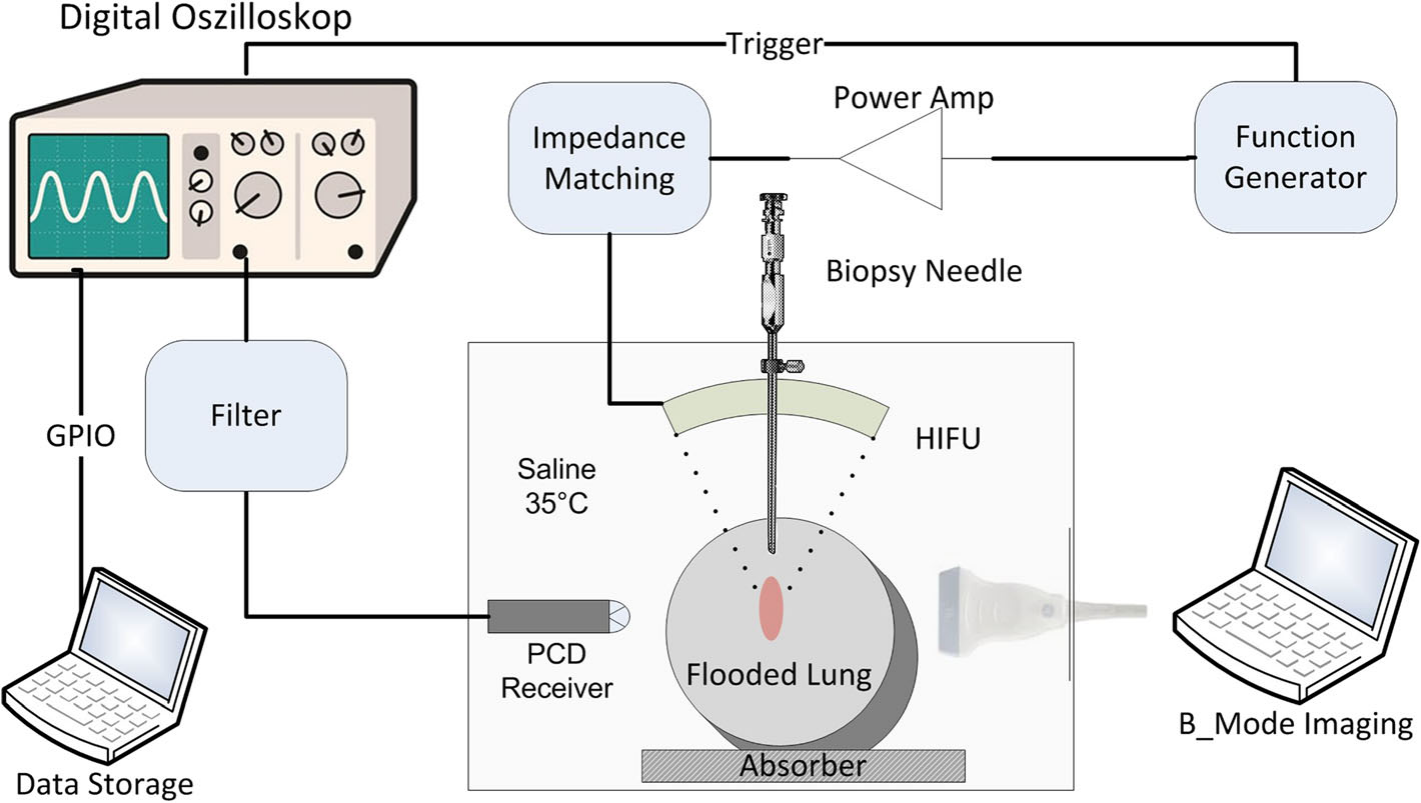
Schematic of the experimental setup for cavitation detection

The experimental setup is shown in Fig. 2. The HIFU transducer (H102, Sonic Concepts, Bothell, WA, USA) with a sideways adjusted imaging probe and PCD detector was immerged in the saline filled tank. The transducer operates at 1,1 MHz, it has an outer diameter of 64 mm and a central opening of 20 mm resulting in an F number of 0,98. The transducer was mounted onto a manual adjustable 3D motion stage, and was driven by a RF power amplifier (RF-Source, Athens, Greece) through the manufacture supplied 50 Ohm impedance matching. Sonographic imaging and HIFU were not synchronised. Signals from the PCD were sampled using a digital oscilloscope (54645A, Agilent, Santa Clara, CA, USA) and transferred via GPIO interface to a PC for data storage. The customized PCD sensor was designed with a 550 kHz centre frequency and moderate 30% bandwidth (Smart Material, Dresden, Germany). The function generator (33120A, Agilent, Santa Clara, CA, USA) and oscilloscope were synchronised using an external trigger.

### HIFU focal intensity calibration

Calibration was performed in a tank filled with degassed water. The setup contained the same HIFU transducer, amplifier and impedance matching as described above. A HIFU membrane hydrophone (HIFU-SI-03, GAMPT, Germany) was placed on a 3 axis motion stage at the focal position. This HIFU compatible hydrophone provides a large bandwidth (100 kHz–100 MHz), high dynamic range, small active element size (0,2 mm), and is able to detect pressure amplitudes up to 100 MPa [17]. The axial and lateral beam profile of the transducer and the maximum focal pressure waveform were characterised, and the hydrophone’s active element was positioned into the centre of the focal spot. The transducer input power was varied in the range of 1,5–288 W. At each power level the received hydrophone waveform was captured and deconvoluted [18] before calculating the temporal peak intensity as follows:

$$ {I}_{SPTP}=\frac{1}{T\ {Z}_{water}}{\displaystyle \sum_t^{t+ T}}{p}^2(t) $$

The resulting intensity and rarefactional pressure functions were derived using second order interpolation. The measured hydrophone amplitudes, derived rarefactional and peak positive pressures as well as the intensity values are summarized in Table 1.

**Table 1:** HIFU focal intensity and pressure table of the HIFU transducer calibration using a membrane hydrophone

Pin [W]	U pp [V]	p rarefact [MPa]	p pos [MPa]	ISPTP [W/cm2]
1,5	0,12	1,0	1,0	38
6,5	0,25	2,1	2,3	161
17,4	0,44	3,5	4,0	416
39,8	0,67	5,0	6,2	976
72,0	0,88	6,1	8,8	1.827
134,5	1,28	8,1	13,2	3.407
196,0	1,27	9,8	19,1	5.291
246,5	2,10	10,9	24,0	7.216
288,0	2,44	12,0	29,2	9.033

### Operational scheme

The focal zone was sonographically targeted into flooded lung parenchyma. Care was taken that no bronchial or cancerous tissue was located in the acoustic path. The HIFU (1,1 MHz) was excited for 2 ms (2200 cycles) with a repetition frequency of 20 Hz. During the HIFU exposure 4000 samples from the PCD sensor signal were acquired at 8 MHz sampling rate. Sonography was initiated manually and stored as a 4 s image frame of 18 Hz framerate. HIFU exposure was applied for 10 s. The focal position was slightly changed when a cavitation event was monitored.

### Tissue sampling

**Fig. 3:**
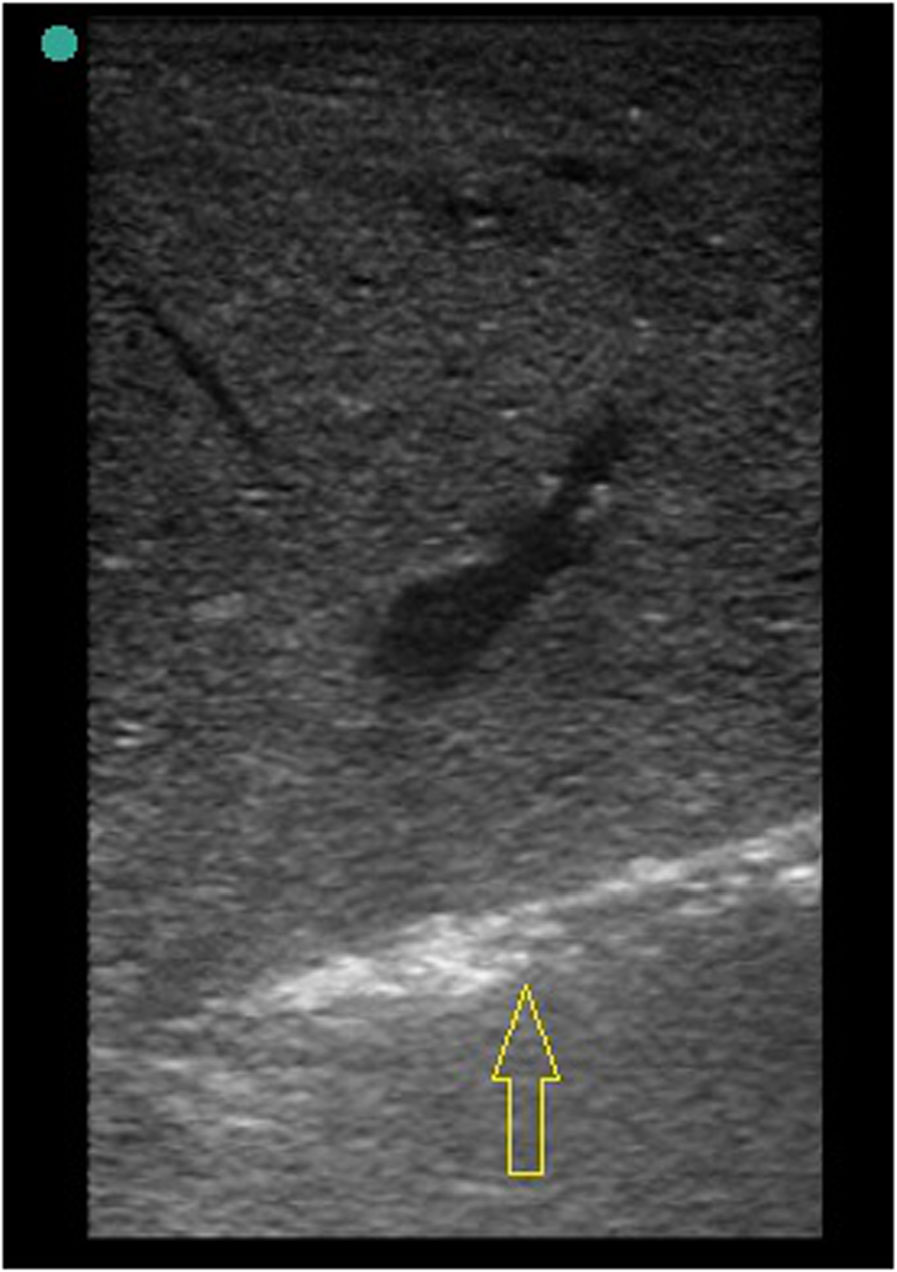
Sonographic image of flooded lung after biopsy shutter release so that tissue is sampled out of the focal zone (*arrow*)

For tissue sampling, a biopsy needle (14 G, Embemed, Germany) was guided through the central opening of the HIFU transducer using a customized conical adaptor. The needle was placed under sonographic guidance, not closer than 1 cm in front of the focal zone. During acoustic measurements, it was made sure that the intensity was unaffected by the needle, so that it did not interfere with the HIFU beam path. Tissue sampling (three per lobe) was performed after 30 s HIFU exposure at an intensity of 4.000 *Wcm* ^‐ 2^(*p*_*r*_ 8, 7 *MPa*) out of the focal zone, and out of non HIFU exposed lung (Fig. 3). Samples were fixed in formalin for histological HE (Haematoxylin and Eosin) staining. All experiments were performed within 45 min after resection.

### Data analysis

Cavitation threshold is defined as a *p* = 0.5 probability of the positive events relative to the number of measurements. The sampled PCD data were analysed using MATLAB (Mathworks, Natwick, USA). The corresponding spectrum of each sample was derived using FFT transformation. A positive passive cavitation event was defined as being when the subharmonic amplitude of the spectrum rose 12 dB above the background level. A sonographically positive event was counted when a cavitation zone appeared visually as a hyperechoic region within one image frame by two independent observers. Threshold of cavitation detection was counted for PCD, sonography as well as for sonographically detected tissue erosion. The probability was interpolated as an error function (ERF) [19].

## Results

**Fig. 4:**
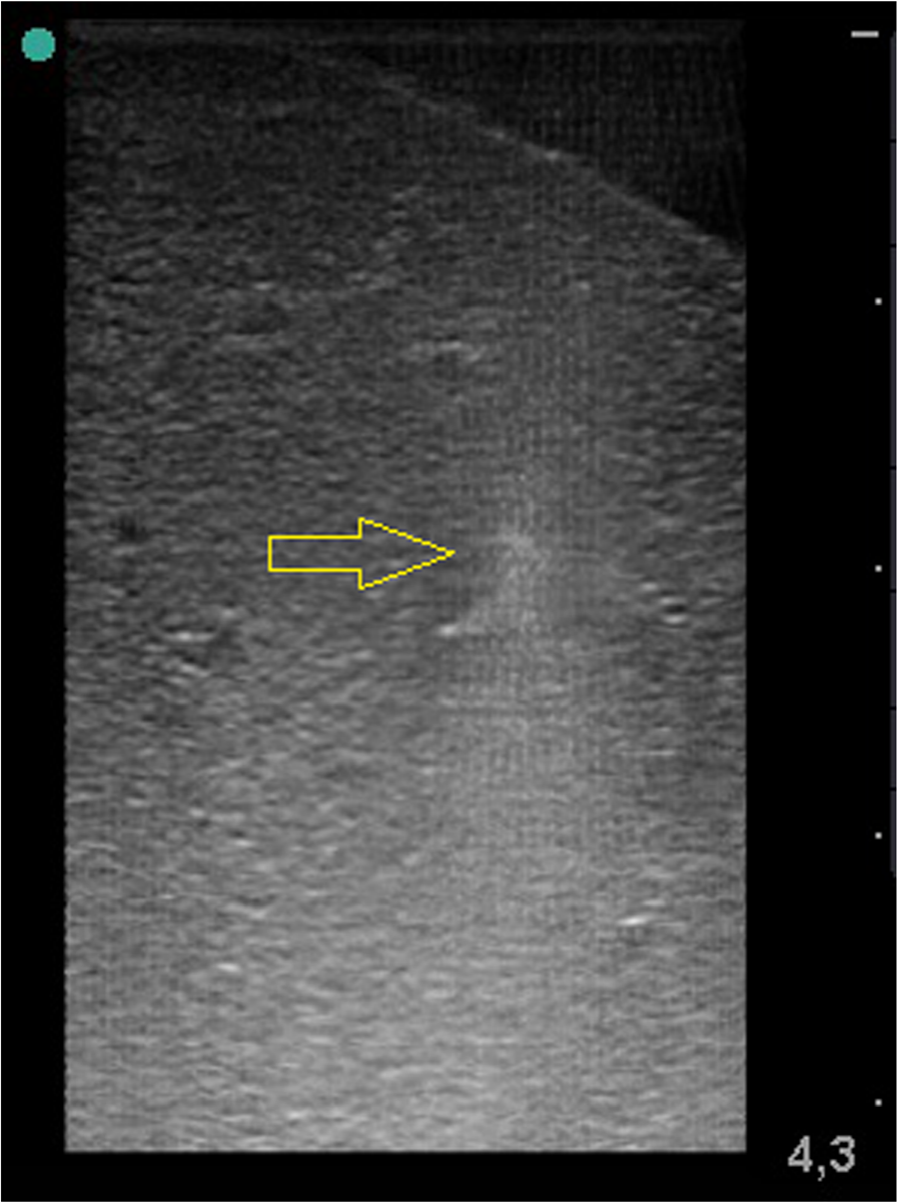
Sonographic manifestation of a cavitation cloud in the focal zone; *arrows* indicate the focal position



Sonographic images revealed the gas free flooding ex-vivo of all lung lobes without remaining gas content. The entire lung lobe was accessible in B mode imaging. Cavitation clouds in the flooded lung appeared as blurred hyper-echoic regions at the focal zone, which were enhanced as intensity increased (Fig. 4). The cavitation zone can be described as inhomogeneous, with stochastic variation of brightness (Additional file 1: Movie S1). After HIFU sonication, the cavitation zone reduced echogenity and disappeared after several seconds.

**Additional file 1: Movie S1.** Sonographic image frame showing positive cavitation events in flooded lung without tissue erosion at 4.000 *Wcm* ^‐ 2^(*p*_*r*_ 8, 7 *MPa*). (AVI 646 kb)


**Fig. 5:**
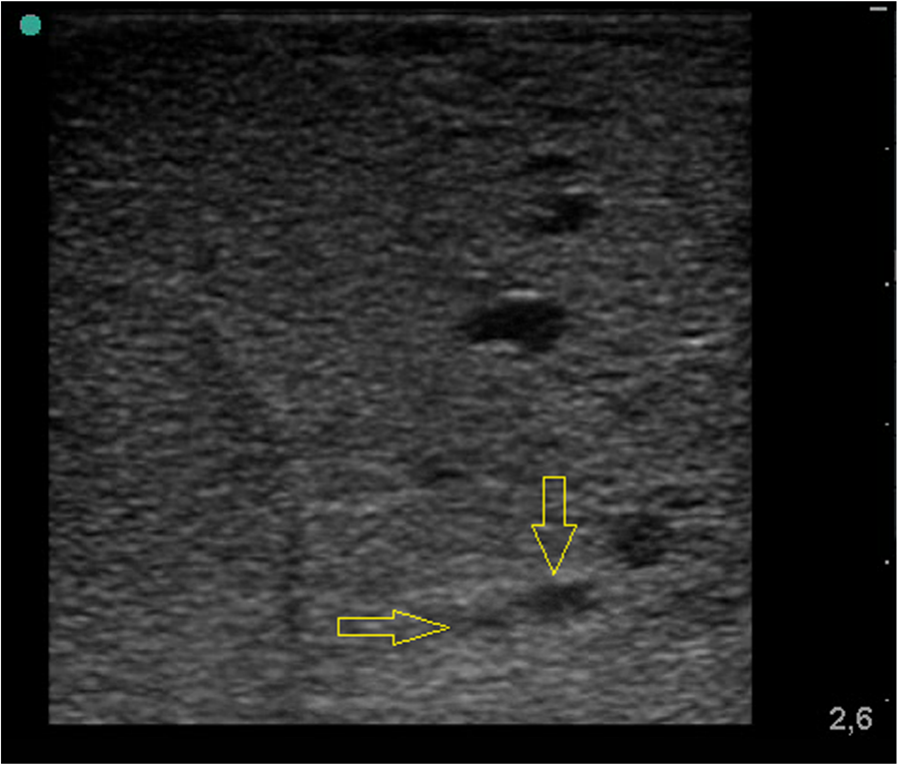
Sonographic image after an erosive cavitation event in flooded lung; *arrow* indicates the focal zone with echoless structure



Focal tissue erosion is characterised by the formation of a hypoechoic lesion in comparison with the pre sonication image. During HIFU exposure, the erosive zone appears mostly hyperechoic with stochastic echogenity and the formation of echoless structures at the focal position (Additional file 2: Movie S2). The entire erosive zone appeared fully visible after the HIFU exposure as a hypo-echoic, cyst like lesion (Fig. 5).

**Additional file 2: Movie S2.** Sonographic image frame during cavitation induced tissue erosion in flooded lung at 9.000 *Wcm* ^− 2^(*p*_*r*_ 12, 0 *MPa*). (AVI 124 kb)


**Fig. 6:**
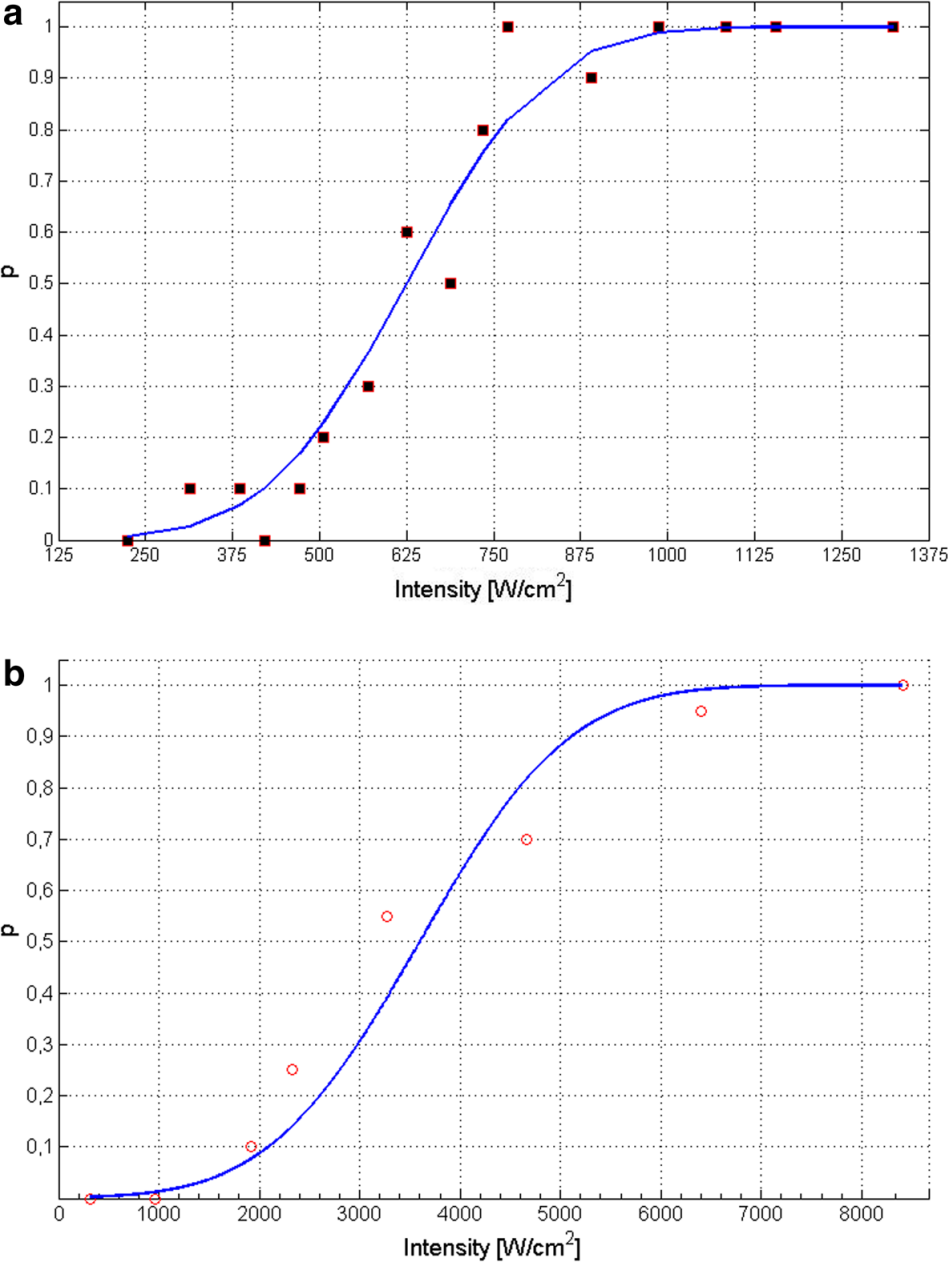
Probability plot for cavitation (**a**) for passive cavitation detection based on subharmonics (PCD) and (**b**) based on sonographic manifestation

The cavitation probability over intensity is shown in Fig. 6a for passive, and in Fig. 6b for sonographic detection. Casual PCD events can be monitored from 350 *Wcm* ^− 2^ (*p*_*r*_ 3, 3 *MPa*), while above 1.000 *Wcm* ^− 2^ (*p*_*r*_ 5, 1 *MPa*) at each measurement a positive cavitation event was detected. A threshold (*p* = 0.5) for PCD based on subharmonics and sonographic manifestation was estimated to be 625 *Wcm* ^− 2^(*p*_*r*_ 4, 3 *MPa*) and 3.600 *Wcm* ^− 2^ (*p*_*r*_ 8, 3 *MPa*), respectively. Lung tissue erosion could be monitored in one lobe at 7.200 *Wcm* ^− 2^(*p*_*r*_ 10, 9 *MPa*), and in two lobes at 9.030 *Wcm* ^− 2^ (*p*_*r*_ 12, 0 *MPa*). Therefore a probability threshold could not be derived (3 out of 7 events) for tissue erosion.

**Fig. 7:**
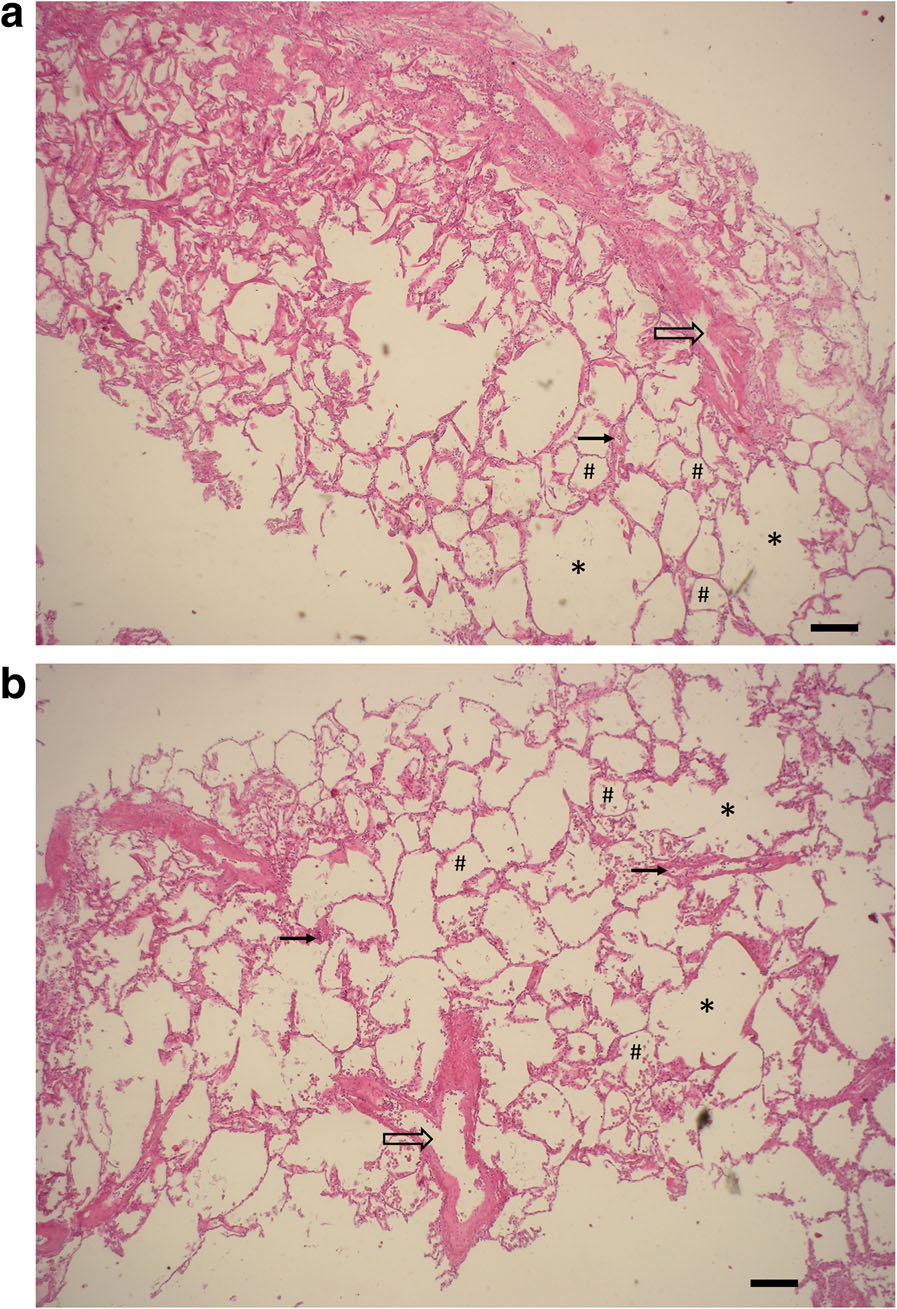
Hematoxylin and eosin stained sections of lung tissue sampled out of (**a**) the HIFU focal zone after exposure with 4.000 Wcm^− 2^(p_r_ 8, 7 MPa) and (**b**) out of non HIFU exposed areas of the same lung. After HIFU exposure no damage of alveolar texture could be detected. Both samples show a mild lung emphysema which is characterized by enlarged air spaces (*) adjacent to normal alveoli (#). *Arrows* mark small vessels (→) and bronchi (⇨). Scale bar, 200 μm; magnification, x40

Histological images of lung parenchyma both after HIFU exposure (Fig. 7a), and when HIFU exposure was absent (Fig. 7b) show no pathological difference. Alveolar tissue was found to be intact in both groups, without disruption of alveoli. In lobes from patients with mild lung emphysema, which is characterised by enlarged airspace, no destruction of alveolar texture occurred after HIFU. The morphological presentation of HIFU and un-exposed lung tissue is similar, without significant damages or micro haemorrhage.

## Discussion

In this study the effects of HIFU induced cavitation has been investigated in flooded lung for the first time. The aim of every HIFU treatment is to ablate cancer tissue, not healthy parenchyma. However in cases of misguiding by motion artefacts or during peripheral exposure at the tumour edge, focal intensities will be exposed to parenchyma as well. Furthermore, in the pre-focal path, minor intensity maxima are also present depending on transducer and beam forming configuration. Those intensities are much lower than in the focal zone, but could also induce cavitation.

As expected, it could be shown that cavitation occurs during HIFU exposure in flooded lung parenchyma. PCD shows a higher detection sensitivity than sonography. It is likely that the hyper-echoic character of flooded lung parenchyma impairs visual sonographic detection. A cavitation cloud is faintly separated from a flooded lung, unlike in echoless targets. In literature, similar conclusions have been found, inasmuch as the sonographic detection of cavitation clouds is less sensitive than PCD [10]. In addition, a movie frame gives the observer a better dynamic visualization than static B-Mode images. HIFU excitation and sonography were not synchronized, as this causes imaging artefacts and therefore worsens the B- Mode cavitation detection.

The cavitation threshold is strongly influenced by temperature. Usually the cavitation threshold reduces during HIFU induced heating. This mechanism is not present in flooded lung, since due to its low attenuation, the HIFU induced temperature rise is much lower than in cancerous tissue [6]. In order to separate the thermal influence from the cavitation process, an excitation scheme with a low duty cycle was used to avoid heating during HIFU exposure.

In our previous work, HIFU was applied into flooded lung parenchyma under temperature monitoring where sonography showed no signs of echo-enhancement and therefore cavitation activity [6]. This finding is conclusive, since the HIFU exposure was performed at 2.400 *Wcm* ^− 2^, which is below the sonographic detection threshold and far below any erosive intensities.

An intensity of 4.000 *Wcm* ^− 2^ for tissue sampling was chosen in this study, which represents a typical intensity for FUS ablation. It is almost twice as high as used in previous studies where we could show the therapeutic effectiveness on lung cancer tissue [4]. At this level sonographic detectable cavitation was induced in flooded lung, that could be used for the targeting of the biopsy needle. The histological stain revealed no morphological damage to the alveolar tissue. The alveolar membrane, vascular and bronchial structures remained intact during HIFU exposure.

It has been well investigated that cavitation can induce erosive effects on tissue. Using sonography, less than 50% of the investigated lung lobes showed erosion events at the highest intensity level. Therefore a statistical threshold could not be derived. The samples with positive erosion events showed clear formation of echoless structures in the location and shape of the focal zone during and after HIFU exposure. It is likely to assume that the cavitation threshold for erosion is slightly above 9.000 *Wcm* ^− 2^(*p*_*r*_ 12, 0 *MPa*). Erosion was only detected in the focal zone, and not in the pre-focal path. In the patient anamnesis from lobes showing erosive events, COPD grade 3–4 with distinct emphysema, and one case of prior radio/chemo therapy was found. This indicates that a chronically impaired lung is more sensitive to HIFU cavitation.

The maximal focal intensity was limited by the specification of the power amplifier, but also close to the recommended highest input power for the HIFU transducer. It is very unlikely that higher intensities will arise during HIFU ablation, except for histotripsy.

Surprisingly, tissue erosion in flooded lung was induced at intensities several times higher than detected passively (625 *Wcm* ^− 2^ *vs*. 7.600 *Wcm* ^− 2^). This finding can be explained by understanding flooded lung as a tissue compound of stiff (alveolar, bronchial) tissue and water. As described by Vlaisavljevich et. al. [20], stiffer tissue (i.e. higher Young’s modulus) is more resistant to erosion than tissue with high water content. Particular in tissue compounds, stronger layers were preserved while weaker tissue eroded [21]. Therefore it can be assumed that cavitation primarily occurs in the water fraction of flooded lung, rather than in the alveolar tissue.

Intensity thresholds for cavitation have been investigated for several tissues, phantom materials and water. Those results do not reflect the special nature of flooded lung as a compound where saline is statically trapped in a dense alveolar structure. Additionally, the saline is likely to be gas saturated from lung tissue and contains dissolved proteins and surfactants. In studies using the same HIFU frequency and scheme, similar thresholds for passive detection were found in both agarose and in-vivo tumours [22] as well as in air saturated water [23]. Erosion in the flooded lung might occur when the size of cavitation bubbles reaches the dimension of the alveolar structure (~100 μm), or if inertial cavitation is induced.

In flooded lung, a therapeutic window exists in which HIFU can be safely applied for cancer ablation before destructive effects on the lung could be expected (2.400 *Wcm* ^− 2^ − 9.000 *Wcm* ^− 2^). The effects of cavitation can be reduced by using a higher HIFU frequency than the applied 1,1 MHz. Since attenuation, and therefore losses in the lung path is low $$ \left(0,12\ \raisebox{1ex}{$ dB$}\!\left/ \!\raisebox{-1ex}{$ cm\  MHz$}\right.\right) $$ [6], higher HIFU frequencies would increase cavitation thresholds.

This study reveals that cavitation occurs in flooded lung. It can be reliable detected passively as well as sonographically. The detection thresholds are below intensities where destructive erosive effects occur. Therefore both methods can be used for cavitation monitoring in order to avoid erosive side effects during HIFU ablation in flooded lung.

The assumption of flooded lung as a saline tissue compound is limited. During flooding, alveolar surfactant might dissolve in saline and influence the surface tension, and therefore decrease the cavitation threshold. To investigate this possibility, the PCD threshold was measured in liquid drained out subsequent to lung flooding (BAL- Broncho Alveolar Lavage Liquid). It showed the same threshold as in saline flooded lung, which indicates that intrapulmonary surfactant does not have an influence on HIFU cavitation in lung. This is conclusive to literature where it has been found that surface tension of BAL is almost identical to water [24].

This study also suffers from some limitations. The focal intensity was derived in free field (water) measurements. The loss during lung penetration was neglected in our study, since the attenuation in flooded lung is rather low.

In this study, the focal zone was targeted only into homogenous flooded lung, however the lung also comprises vascular and bronchial structure. The effects of HIFU on vasculature has been studied [12, 25], but not investigated to date on large bronchial structures. Erosive effects of HIFU cavitation on a bronchus have not been seen in this study. But it can be assumed that stiff bronchial structure is more resistant to cavitation than alveolar tissue.

In the future, *in-vivo* experiments should be conducted to investigate the physiological response of lung parenchyma exposed to HIFU induced cavitation on large animal trails.

## Conclusions

Within this study, the effects of HIFU cavitation on flooded lung parenchyma has been investigated for the first time. It could be shown that intrapulmonary cavitation can be detected passively as well as sonographically. A therapeutic window exists where HIFU ablation of lung cancers can be safely performed without possible parenchymal erosion in cases of misguiding or in the pre-focal path.

## Abbreviations

*I*_*SPTP*_: Focal Intensity spatial - temporal peak
*Z*_*water*_: Impedance of water
*p*_*pos*_: Positive peak pressure
*p*_*r*_: Negative peak pressure
BAL: Broncho Alveolar Lavage
COPD: Chronic obstructive pulmonary disease
HIFU: High intensity focused ultrasound
OLF: One-Lung Flooding
PCD: Passive cavitation detection
*T*: Period of an acoustic wave
*p (t)*: Acoustic pressure
